# Gut Microbiota and Mental Health: A Comprehensive Review of Gut-Brain Interactions in Mood Disorders

**DOI:** 10.7759/cureus.81447

**Published:** 2025-03-30

**Authors:** Ishani Mehta, Keshav Juneja, Tharun Nimmakayala, Lajpat Bansal, Shivani Pulekar, Dileep Duggineni, Hana Khan Ghori, Nishi Modi, Salma Younas

**Affiliations:** 1 Psychiatry and Behavioral Sciences, Maharaja Agrasen Institute of Medical Research and Education, Hisar, IND; 2 Psychiatry, BJ Medical College, Ahmedabad, IND; 3 Medicine and Surgery, Apollo Institute of Medical Sciences and Research, Chittoor, IND; 4 General Practice, Davao Medical School Foundation, Davao, PHL; 5 Internal Medicine, Siddhartha Medical College, Vijayawada, IND; 6 Internal Medicine, JSS Medical College, Mysore, IND; 7 Medicine, Government Medical College, Surat, Surat, IND; 8 Pharmacy, Punjab University College of Pharmacy, Lahore, PAK

**Keywords:** anxiety, blood-brain barrier, depression, dysbiosis, gut-brain axis, mental health, microbiota, mood disorders, neurotransmitters, short-chain fatty acids

## Abstract

The human gut flora of trillions of bacteria is vital for general health and greatly influences digestion, immune system function, and brain development. Through neuronal, hormonal, and immunological channels, the gut-brain axis (GBA), a bidirectional communication network, links the gut microbiota to the central nervous system (CNS). This relationship has been linked to affective diseases, including depression and anxiety, as well as mental health issues.

This review explores the intricate relationship between gut bacteria and mood disorders, focusing on how gut microbiota-host interactions, immune system modulation, and neurotransmitter control support mental health. The function of important microbial metabolites, including short-chain fatty acids (SCFAs), in preserving blood-brain barrier integrity and modulating neuroinflammation is covered in this review. It also examines the bidirectional impact between gut health and mental health, including how dysbiosis could aggravate mood disorders and how depressed states might change the composition of gut bacteria. Furthermore, we discuss how psychotropic drugs affect gut flora and consider other elements such as nutrition and lifestyle that affect gut microbiome composition. Potential paths for treating mood disorders through gut microbiota modification are presented as emerging treatment techniques, including probiotics, nutritional therapies, and precision medicine.

The development of new therapeutic approaches for mood disorders depends on the awareness of the GBA. Gut bacteria significantly affect mental health through immune modulation, neurotransmitter generation, and other intricate processes. Future studies should concentrate on large, varied populations to better understand these interactions and to create customized treatments that combine gut microbiota modulation with conventional mental health therapies.

## Introduction and background

The human gut microbiota, composed of trillions of microorganisms, is crucial for overall health, impacting digestion, immune function, and brain health [1]. Recent research has highlighted the gut-brain axis (GBA), a communication network between the gut microbiota and the central nervous system (CNS) through neural, hormonal, and immune pathways, suggesting that the gut microbiota can significantly impact brain function and mental health [2].

Mood disorders such as depression and anxiety are prevalent globally and impose substantial societal and economic burdens [3]. Emerging evidence indicates that the gut microbiota may play a crucial role in mood disorders by affecting neurotransmitter systems (chemical messengers in the brain, such as serotonin and dopamine), immune responses, and stress mechanisms (such as the hypothalamic-pituitary-adrenal (HPA) axis) [4]. The GBA theory proposes that the gut microbiota influences brain function through mechanisms such as the vagus nerve (a major nerve connecting the brain and gut, facilitating direct communication), neurotransmitter production, and immune response regulation [5].

Studies have shown that microbial metabolites such as short-chain fatty acids (SCFAs) (small molecules produced when gut bacteria ferment dietary fiber) affect brain function by altering inflammation and neurotransmitter production [6].

Both clinical and preclinical studies have shown a bidirectional relationship between gut health and mental health, indicating that changes in the gut microbiota can affect mood and behavior, and vice versa. Recent research has advanced our understanding of this link using animal models (laboratory experiments using rodents to study gut-brain interactions) and human clinical trials (research studies involving human participants to test interventions) [7]. Limitations such as small sample sizes, short study durations, and diverse study designs persist and necessitate further research. Our understanding remains incomplete, especially regarding diverse study populations and gut-brain interaction mechanisms [8].

Given these findings, modulating gut microbiota through probiotics, prebiotics, diet, and fecal microbiota transplantation (FMT) is emerging as a potential therapeutic strategy for mental health conditions. Several clinical studies suggest that interventions targeting gut microbiota may alleviate symptoms of depression and anxiety, highlighting the potential for microbiome-based treatments in psychiatric care. However, challenges such as individual variability in microbiome composition, limited large-scale trials, and regulatory concerns must be addressed before these interventions can be widely implemented in clinical practice.

This review aims to synthesize current evidence linking gut microbiota to mood disorders, identify gaps in the literature, and suggest future research directions. It explores microbiota-host interactions, diet, microbial metabolites, and psychotropic medication effects (the influence of antidepressants and antipsychotics on gut microbiota) to provide a detailed view of the gut-brain relationship.

## Review

Microbiota-host interaction and brain

The GBA is a bidirectional network that connects the CNS with the enteric nervous system (ENS) in the gastrointestinal (GI) tract. ENS is a network of neurons in the GI tract that controls digestive processes independently of the CNS. It involves neural, hormonal, and immunological pathways. The GBA plays a crucial role in physiological processes like digestion, metabolism, mood, and cognition. Dysfunction in this axis is linked to conditions like irritable bowel syndrome (IBS), depression, anxiety, and neurodegenerative disease, highlighting its potential for novel treatments. Understanding gut-brain connections offers the potential for novel treatments for related disorders.

Pathways of Interaction

Immune system: The immune system crucially links the gut and the brain, coordinating complex interactions. Gut bacteria activate the innate immune system via pattern recognition receptors (PRRs: immune system proteins that detect microbial components, triggering immune responses), such as Toll-like receptors (TLRs: a type of PRR that identifies pathogens and activates immune signaling pathways), on intestinal cells [9]. This triggers the release of cytokines, such as TNF-α, IL-1, and IL-6. These cytokines can cross the blood-brain barrier (BBB) and affect brain function by altering neurotransmitter metabolism, including gamma-aminobutyric acid (GABA), serotonin, and dopamine, which are essential for mood and cognitive functions [10,11]. Immune activation and disruption of neurotransmitter levels can exacerbate neuropsychiatric disorders like depression and anxiety. Inflammation can also compromise the intestinal barrier, increase gut permeability, and intensify inflammation and immune response [12]. The vagus nerve facilitates bidirectional communication between the gut and the brain. Gut immune activity activates vagal afferents that signal the brain, whereas brain signals regulate gut immune responses via the cholinergic anti-inflammatory pathway [13]. This interaction influences the autonomic nervous system (ANS) and HPA axis (a hormonal system regulating stress responses, involving the hypothalamus, pituitary gland, and adrenal glands), modulating stress responses and creating a feedback loop between stress and immunity [14]. Imbalances in immune signaling within the GBA are linked to diseases such as Parkinson's and Alzheimer's through neuroinflammation and neuronal damage [15]. Dysbiosis, an imbalance in gut microbiota composition, can affect immune function, digestion, and mental health. Understanding these immunological interactions is critical for designing medicines that restore GBA balance and treat associated diseases.

**Figure 1:**
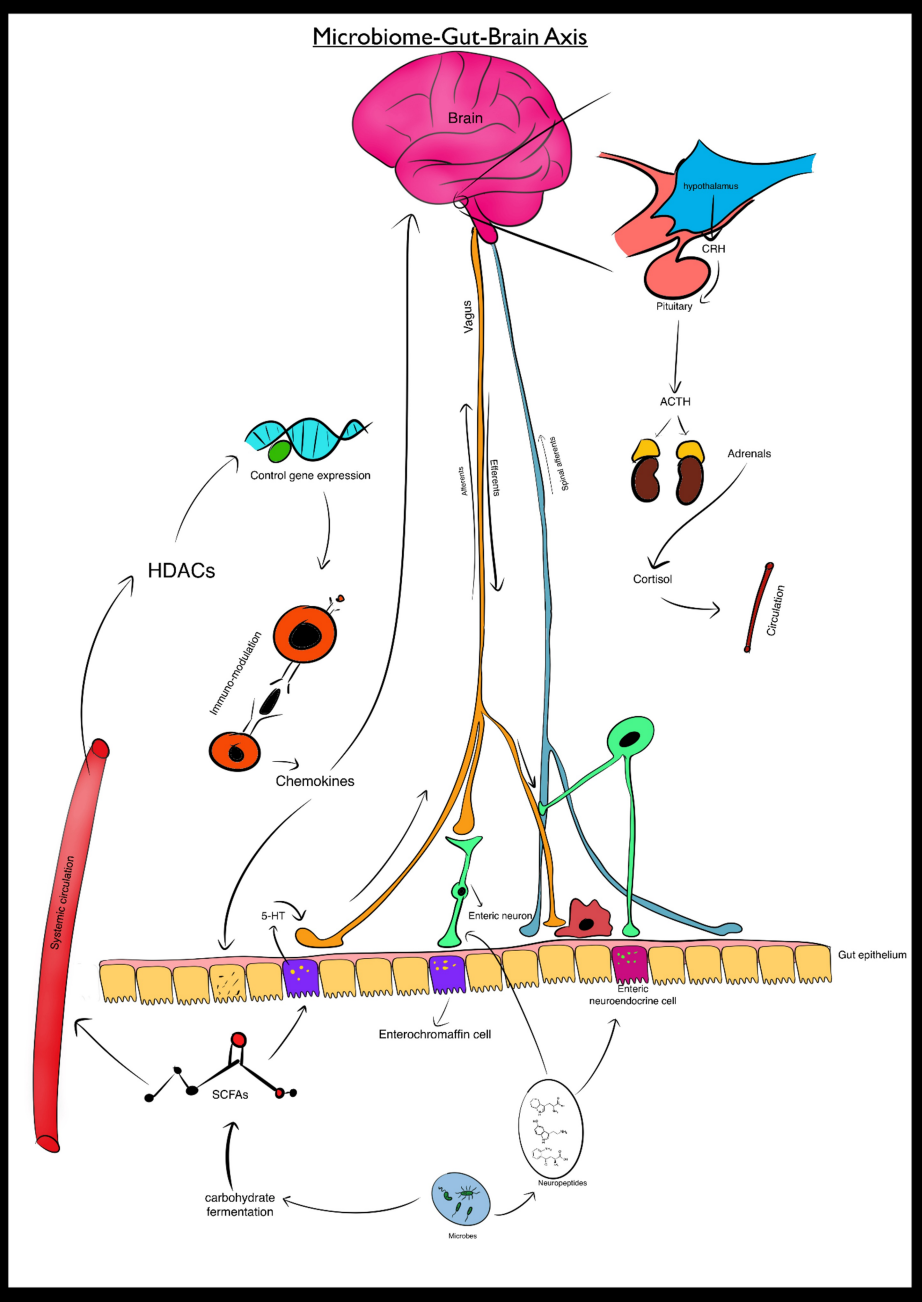
Microbiome-Gut-Brain Axis: Bidirectional Communication Pathways Schematic representation of the microbiome-gut-brain axis. The diagram shows the bidirectional communication between the gut and the brain through neural, hormonal, and immune pathways, emphasizing the role of gut microbiota in modulating brain function and mental health. HDACs: histone deacetylases; 5-HT: 5-hydroxytryptamine; SCFAs: short-chain fatty acids; CRH: corticotropin-releasing hormone; ACTH: adrenocorticotropic hormone Credit: Image created by the author

Vagus nerve: The vagus nerve, or tenth cranial nerve (CN X), is the longest cranial nerve, extending from the brainstem to the heart, lungs, and GI tract. The vagus nerve is an intricate network of motor and sensory fibers that controls various physiological processes. It plays a crucial role in the GBA by transmitting sensory information from the gut to the brainstem. Receptors in the GI tract detect changes and send these data to the brainstem via vagal afferent fibers [16]. This information is processed in the nucleus tractus solitarius (NTS) and then relayed to higher brain regions, allowing for the perception of gut sensations and appropriate responses [17]. The nerve also controls motor functions in the digestive system, including GI motility, secretion, and absorption, through its influence on smooth muscles, glands, and enteric neurons [18]. The vagus nerve regulates the ENS and neuroimmune functions, maintains immune balance, and limits inflammation through acetylcholine [19]. Vagus nerve stimulation (VNS) has the potential to treat these conditions by restoring the vagal tone and modulating neuroimmune interactions. Figure 1 visually represents the complex interactions between the gut microbiota and the brain, specifically highlighting the GBA.

Microglia and BBB: Microglia are specialized immune cells in the CNS that protect against infections and remove damaged neurons and debris. Research indicates that microbial metabolites from the gut can influence microglial activation via pathways involving cytokines, neurotransmitters, and neurotrophic factors [20]. The interaction between microglia and gut bacteria is key for regulating neuroinflammation, synaptic plasticity (the ability of neurons (nerve cells) to strengthen or weaken their connections over time, which is essential for learning and memory), and neuronal balance, thus affecting behavior and brain function. The BBB acts as a selective barrier between the bloodstream and the CNS but can also facilitate the transfer of gut-derived signals, including immune mediators and microbial metabolites, into the brain, indicating its role in gut-brain communication [21]. Gut-derived signals can increase BBB permeability through inflammatory mediators, allowing proinflammatory substances to enter the brain, which may lead to neuroinflammation and neurological conditions. Conversely, beneficial gut metabolites, such as SCFAs, protect the BBB and reduce neuroinflammation [20,22], whereas gut chemicals also influence BBB permeability and immune cell activity. Gut-derived chemicals can affect BBB permeability and neuroinflammation by altering peripheral immune cell activity, including that of T cells and monocytes [23].

Role of Neurotransmitters

Gut microbiota plays a vital role in modulating various neurotransmitters, thereby optimizing mood and cognitive function through several complex mechanisms. The synthesis of neurotransmitters or their precursors, which can cross the BBB and contribute to neurotransmitter production in the brain, is facilitated by enzymes released by gut bacteria [24]. Gut microorganisms produce metabolites that signal intestinal enteroendocrine cells to produce and release neurotransmitters. These neurotransmitters can act locally in the ENS or transmit signals to the brain via the vagus nerve [25]. Gut bacteria affect neurotransmitter levels by modulating the immune system and producing anti-inflammatory metabolites like SCFAs, which can indirectly influence brain health [26].

Gut microbiota affects neurotransmitter balance and influences mental health and cognition. Gut bacteria interact with the CNS through neurotransmitters like GABA, dopamine, norepinephrine, serotonin, and histamine. Serotonin is a neurotransmitter found in both the central and peripheral nervous systems, with over 90% of the gut regulating peristalsis, pain, nausea, and secretion. It is produced by enterochromaffin cells using the enzyme tryptophan hydroxylase 1 (TpH1), which converts tryptophan to serotonin [27]. Gut microbiota regulates TpH1 in enterochromaffin cells, boosting serotonin levels in the gut [28]. While peripheral serotonin cannot cross the BBB, the gut microbiota influences central serotonin by altering tryptophan metabolism, affecting its levels in the blood and its conversion to serotonin in the CNS [29].

Dopamine, a crucial neurotransmitter that plays crucial roles in mood, motivation, and motor control, is produced in brain regions such as the substantia nigra and ventral tegmental areas. Dysregulation of dopamine levels is linked to neurological and psychiatric disorders such as Parkinson’s disease, schizophrenia, and addiction [30-32]. In the gut, bacterial strains like *Staphylococcus *can produce dopamine from levodopa (L-DOPA) with the help of staphylococcal aromatic amino acid decarboxylase (SadA), affecting gastric secretion, blood flow, and motility [25].

GABA, the primary inhibitory neurotransmitter in the CNS, helps to balance neuronal excitation and inhibition, regulates muscle tone, has a calming effect on the brain, and controls anxiety, stress, and fear. GABA is synthesized from glutamate by the enzyme glutamate decarboxylase, which is linked to several neurological and psychiatric disorders such as epilepsy, anxiety disorders, and depression [33,34]. Evidence has shown that GABA is produced by various microorganisms, and germ-free animals show reduced peripheral GABA levels, highlighting the role of the gut microbiota [35]. Although GABA from gut microbes typically does not directly cross the BBB, microbiota-derived metabolites, such as acetate, can traverse the barrier and may influence GABA metabolism in the CNS, with mechanisms still being explored [24].

Norepinephrine, a neurotransmitter and hormone crucial for regulating arousal, alertness, and the fight-or-flight response, is synthesized in the locus coeruleus and adrenal medulla. It also affects cardiovascular functions, such as heart rate and blood pressure, and cognitive processes, such as attention and memory consolidation. Dysregulation is linked to various conditions, including anxiety, Parkinson's disease, and hypertension [36]. In vivo studies have shown that certain bacteria can produce norepinephrine, with reduced levels in germ-free mice and rising levels after microbiota colonization, although whether this is due to direct bacterial production or indirect effects remains unclear [37,38]. The microbiota significantly affects the catecholamine systems. For instance, antibiotic-treated mice showed heightened cocaine sensitivity, which was normalized by SCFAs from microbial fermentation, suggesting an indirect impact on norepinephrine [39].

Impacts of microbial metabolites

Gut microbiota produce key neurotransmitters involved in mood regulation. Lactobacilli secrete acetylcholine and GABA, whereas *Candida*, *Streptococcus*, *Escherichia*, and *Enterococcus *secrete serotonin. *Bacillus *and *Serratia *secrete dopamine [40]. These neurotransmitters play essential roles in emotion and mood regulation [41].

SCFAs, including acetate, propionate, and butyrate, are produced by gut microbiota via carbohydrate fermentation [42]. SCFAs cross the BBB through monocarboxylate transporters (MCT) and influence the immune system and gene expression via free fatty acid receptors and histone crotonylation [42,43]. Low SCFA levels are associated with major depressive disorders, and SCFA supplementation reduces depressive behaviors in mice, although human studies are less conclusive [44,45].

Brain-derived neurotrophic factor (BDNF) is critical in the GBA. In mice administered antibiotics, gut dysbiosis leads to altered BDNF levels in the hippocampus and amygdala, correlating with behavioral changes [46].

Tryptophan, GABA, and dopamine are vital for mood modulation. Tryptophan is a serotonin precursor, and its metabolites, such as kynurenine, affect pro-inflammatory cytokines and are linked to mood disorders like postpartum depression [47]. GABA, produced by *Bacteroides*, *Parabacteroides*, and *Escherichia*, acts as both an excitatory and inhibitory neurotransmitter, and its modulation influences depressive behavior [48]. Dopamine, produced by gut microbes such as *Lactobacillus *and *Bacillus*, is essential for reward-related behaviors; however, the role of gut-derived DA in mood regulation requires further exploration [49]. Nitric oxide (NO), produced by gut microbiota through anaerobic respiration, is associated with suicidality, and bacterial metabolites such as indole downregulate NO production and reduce neuroinflammation [50,51].

Hydrogen sulfide, produced by gut microbes via cysteine degradation, affects synaptic plasticity, a key element in mood disorders [52]. Decreased hydrogen sulfide levels are linked to depression severity, making it a potential therapeutic target [53]. Carbon monoxide (CO), produced by the microbiota through heme oxygenase 1 (HO1), has antidepressant effects in animal studies by increasing dopamine and affecting heme oxygenase activity in the hippocampus [54].

The gut microbiota synthesizes various polyamines such as spermine, spermidine, and putrescine in the presence of amino acid precursors [55]. In humans, putrescine is produced in the cytoplasm of cells by decarboxylation of ornithine catalyzed by the enzymes ornithine decarboxylase (ODC), spermine, and spermidine, which are synthesized by S-adenosyl-methionine decarboxylase [56]. The brain exhibits a cellular stress response referred to as the polyamine-stress response (PSR) when exposed to stressful stimuli, with an initial, brief elevation in polyamine metabolism [56]. The magnitude of the response was found to correlate with the intensity of the stress; however, in the presence of chronic stress, a partial response was observed, leading to the accumulation of putrescine and reduced levels of higher polyamines such as spermidine and spermine [56,57]. Long-term inhibition of polyamine synthesis due to chronic stress has been found to deplete brain polyamines and alter emotional reactivity to stressors [58]. Decreased levels of polyamines are found in the hippocampus and accumbens septi in a rat model, and low plasma levels of agmatine are observed in depression, which is normalized to treatment [58]. S-adenosyl methionine, vital for the production of polyamines, has been used to treat depression in humans [59].

**Figure 2:**
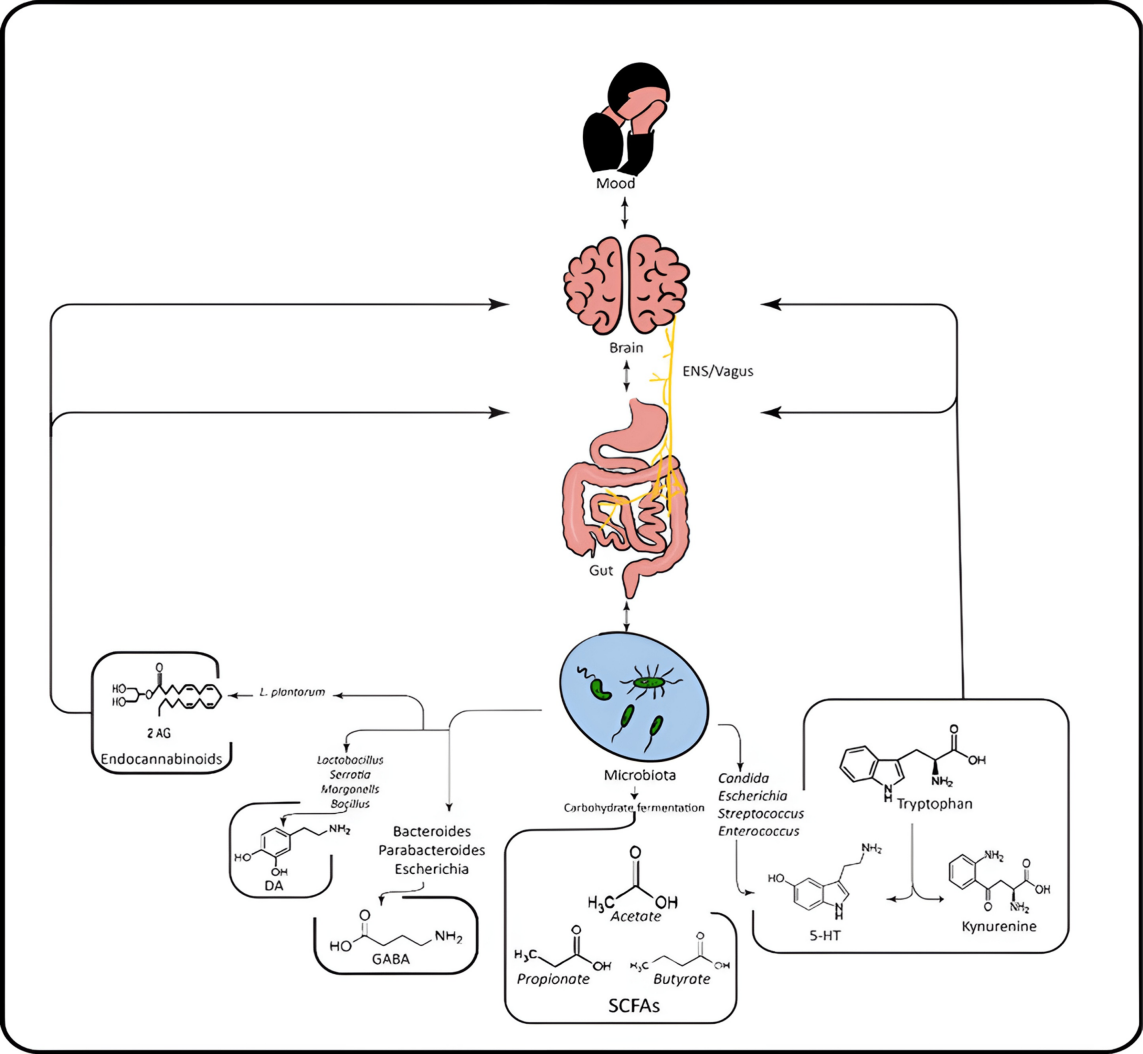
Gut Microbiota, Neurotransmitters, and Mood Regulation The interplay between gut microbiota, neurotransmitters, and mood. This figure illustrates how gut-derived neurotransmitters and metabolites influence mood regulation through the gut-brain axis. ENS: enteric nervous system; vagus: vagus nerve; SCFAs: short-chain fatty acids; 5-HT: 5-hydroxytryptamine; DA: dopamine; GABA: gamma-aminobutyric acid; 2-AG: 2-arachidonoylglycerol Credit: Image created by the author

Figure 2 highlights the specific interactions between gut microbiota, neurotransmitters, and mood regulation.

Depression and gut microbiome

The relationship between the gut microbiota and depression is bidirectional and complex and involves multiple physiological pathways. Emerging research highlights how changes in gut microbiota composition can influence depressive symptoms and vice versa.

Gut microbiota can affect brain function and mood regulation through various mechanisms. Several studies have shown that an imbalance in gut microbiota, known as dysbiosis, is associated with depressive disorders [60]. Dysbiosis can lead to increased intestinal permeability, commonly referred to as "leaky gut," allowing bacterial endotoxins such as lipopolysaccharides (LPS) to enter the bloodstream. Leaky gut is a condition in which the intestinal lining becomes too porous, allowing harmful substances to enter the bloodstream [61]. This triggers systemic inflammation, a well-known factor in the development of depression. Elevated inflammatory markers, such as C-reactive protein (CRP) and pro-inflammatory cytokines (e.g., IL-6 and tumor necrosis factor-alpha (TNF-α)), have been consistently observed in individuals with depression [62]. Moreover, SCFAs produced by gut microbiota, such as butyrate, propionate, and acetate, play a role in maintaining the integrity of the BBB, reducing neuroinflammation, and influencing the production of neurotrophic factors like BDNF, which supports neuronal growth and function [63].

In contrast, depressive states can also lead to changes in gut microbiota composition. Psychological stress and depression can modify eating habits, reduce gut motility, and alter the secretion of digestive enzymes and mucus, all of which affect the gut microbial ecology [64]. Chronic stress, which is common in depressive disorders, activates the HPA axis and increases cortisol levels. Cortisol can alter gut permeability and affect gut microbiota composition, reducing beneficial bacteria such as *Lactobacilli *and *Bifidobacteria *while promoting the growth of harmful bacteria [65]. Additionally, depressive behaviors, such as changes in diet and reduced physical activity, can lead to an altered gut microbiota profile. For instance, diets high in fat and sugar, often associated with depression, can promote dysbiosis and further worsen depressive symptoms [66].

Mechanisms

**Figure 3:**
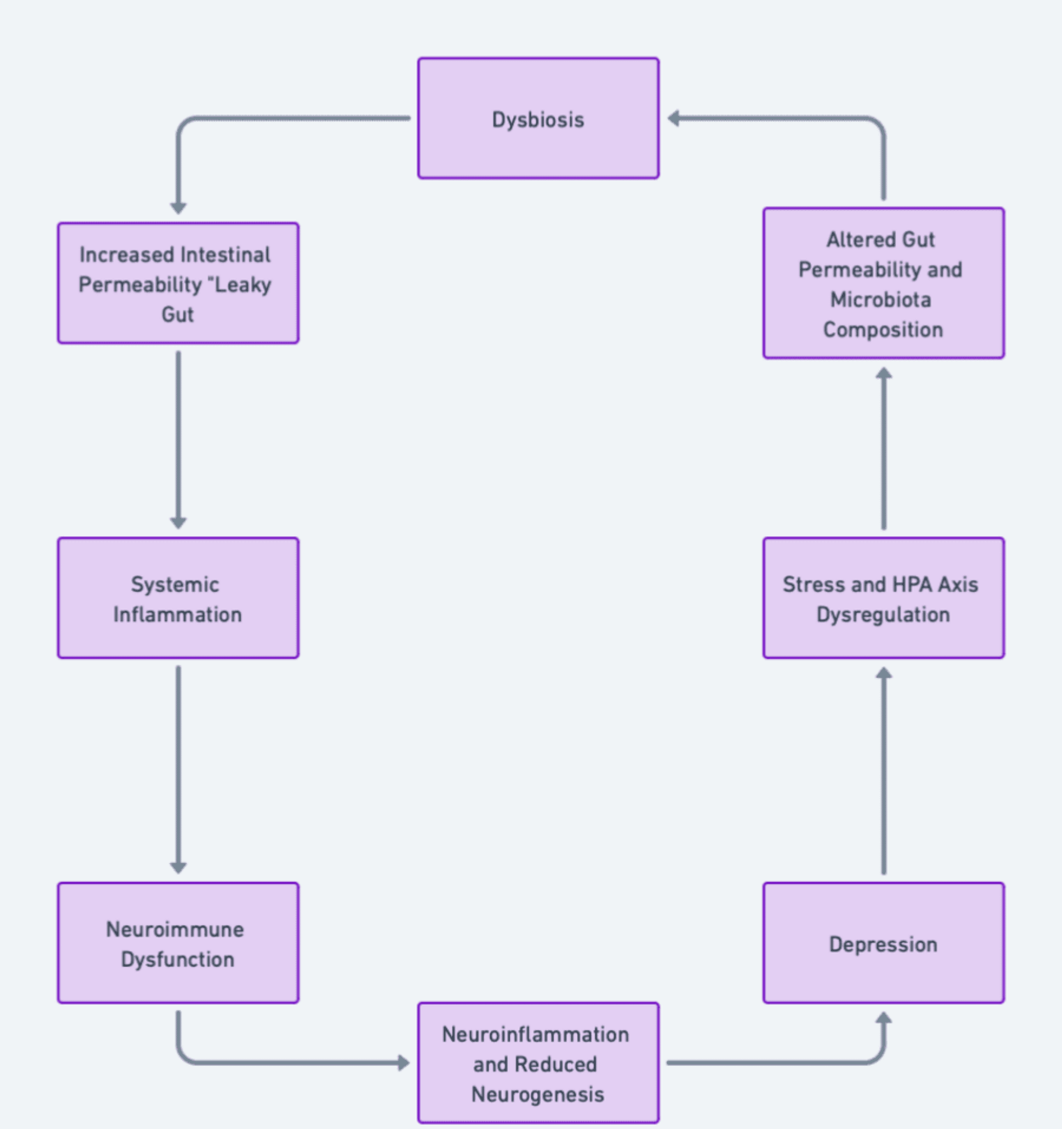
Dysbiosis, Intestinal Permeability, and Depression: Pathway Overview Pathway illustrating the relationship between dysbiosis, intestinal permeability, and depression. The figure shows how gut microbiota imbalance can lead to systemic inflammation, neuroimmune dysfunction, and ultimately depressive symptoms. Credit: Image created by the author

The mechanisms underlying the gut microbiota-depression relationship primarily involve neuroimmune dysfunction and HPA axis dysregulation. Dysbiosis-induced inflammation is a key player in the neuroimmune pathway. Gut-derived inflammatory mediators can cross the BBB and activate microglia and resident immune cells of the brain. This activation leads to neuroinflammation, which disrupts neurogenesis and synaptic plasticity and contributes to the development of depression [67]. The HPA axis, a central stress response system, is often dysregulated in patients with depression. The gut microbiota can modulate the HPA axis through the production of microbial metabolites that influence cortisol levels. Dysbiosis can lead to an exaggerated HPA axis response to stress, resulting in elevated cortisol levels, which further perpetuate gut permeability and inflammation, thereby creating a vicious cycle [68]. Figure 3 illustrates how gut dysbiosis can lead to depression via various physiological pathways.

Bipolar disorder (BD) and gut microbiome

BD is a severe, chronic, and recurring mood disorder classified as bipolar I and bipolar II. While bipolar I entails at least one manic episode, bipolar II is characterized by both hypomania and depressive symptoms [69]. The mechanisms by which the gut microbiota affects the pathogenesis of BD are multifold, with one of them being through immune dysregulation [70].

The association between BD, gut microbiota, and immune response can be presumed by acknowledging the correlation between BD and gut inflammatory diseases like IBD. For instance, the diagnostic panel for Crohn's disease includes detecting antibodies against *Saccharomyces cerevisiae*, a normal gut microbiota organism. Notably, patients also often exhibit elevated levels of antibodies against *S. cerevisiae*, particularly near the onset of the disease. It is likely that the presence of these antibodies indicates a response to the yeast’s presence at the mucous-blood interface, which is disrupted during inflammation [71,72].

In this context, it is important to consider the potential role of antibiotic therapy in triggering acute manic episodes in patients with BD [73]. A study of patients hospitalized for acute mania found a significant link between recent antibiotic use and more severe manic symptoms [74]. It should be noted that a hypothesis suggests that antibiotics can alter gut bacteria, which may affect proteins that regulate the BBB, as well as cytokines, BDNF, and 5-hydroxytryptamine (5-HT) transporters. These changes could lead to cognitive impairment over time [75].

Some studies have identified an association between the bacteria *Flavonifractor*, which degrade the antioxidant quercetin, and BD [76,77], particularly in female smokers. These bacteria may elevate oxidative stress and inflammation by disrupting quercetin production. This finding aligns with earlier studies that reported increased oxidative stress and low-grade inflammation in BD patients [78,79].

Immune dysfunction in BD may be influenced by a complex interplay between stress, gut microbiota, genetics, and epigenetics [80]. Intriguingly, gut microbiome diversity and consistency were negatively correlated with the methylation status of the aryl hydrocarbon receptor nuclear translocator-like gene (ARNTL gene), which is known to regulate monoamine oxidase transcription. Recent studies have proposed that changes in the overall gut microbiome diversity could act as an internal environmental factor, influencing the epigenetics of the ARNTL clock gene, which is implicated in BD development [81].

The pathogenic role of gut microbiota in neuroinflammation is seen in BD. A recent study, consisting of medication-free BD patients with depression episodes using a mouse model with FMT transplantation, provided a plausible association between gut microbiota and neuroinflammation [82].

Gut microbial dysbiosis has been linked to compromised BBB integrity and increased permeability [83,84], which are implicated in neuropsychiatric disorders [85]. A compromised BBB may facilitate the entry of gut microbe-derived components such as LPS into the CNS, triggering neuroinflammation. This LPS stimulation can induce microglial transformation into the M1 phenotype and provoke the release of proinflammatory cytokines (IL-1, IL-6, TNF-α), thereby upregulating TRANK1 expression in neurons [86]. TRANK1 overexpression leads to reduced dendritic spine density in cortical neurons.

This loss of dendritic spine density in the prefrontal cortex has been implicated in the cognitive deficits and behavioral abnormalities seen in BD [84,87-89]. Research also indicates that individuals with BD exhibit a developmental defect in synaptic pruning, resulting in abnormalities in the modulation of neuronal connectivity within the ventral prefrontal and limbic cortices. Synaptic pruning is a natural process where the brain removes excess synaptic connections during development; abnormalities in pruning are linked to psychiatric disorders [90,91].

KAT-2 is an essential enzyme that synthesizes kynurenic acid (KYNA) from kynurenine (KYN), a process central to the tryptophan metabolic pathway [92]. Tryptophan catabolism can be triggered by inflammatory stimuli such as viral infections, bacterial LPS, and interferon stimulation [93]. In patients with BD, elevated CSF KYNA levels have been reported in previous studies and linked to manic episodes and psychotic symptoms [94,95]. These findings suggest that dysregulation of the KYN-KYNA metabolic pathway may be involved in BD pathophysiology [84].

The Interaction Between GI Hormones, Gut Bacteria, and Cognitive Impairment in BD

Glucagon-like peptide-1 (GLP-1) signaling plays a neuroprotective role by influencing synaptic plasticity and neuroinflammation and enhancing cognitive functions such as learning, memory, executive function, and attention [96,97]. GLP-1 receptors are found in key brain regions such as the cerebral cortex, hypothalamus, and limbic system, which play important roles in regulating emotions and cognition [98]. Thus, diminished GLP-1R signaling due to decreased levels of butyrate-producing bacteria in BD patients might compromise synaptic plasticity and cognitive function, as evidenced by multiple studies [99].

Another GI hormone, peptide YY (PYY), is a satiety hormone produced by L-cells in the intestines, influencing the CNS by suppressing appetite-stimulating neurons that produce neuropeptide Y [100]. While direct evidence linking PYY to BD is scarce, lower peripheral PYY levels could potentially contribute to reduced GABA inhibition. This is noteworthy as both neuropeptide Y and GABA are released by neurons in the arcuate nucleus. These reduced GABA levels could be potentially linked to cognitive impairment in BD [101,102].

Effects of psychotropic medications on gut microbiota

Psychotropic medications, typically used for managing mental disorders, interact bidirectionally with the gut microbiota, meaning that they can both affect and be affected by these microorganisms [103-109]. Commonly used psychotropic drugs for mood disorders include antipsychotics, antidepressants, anticonvulsants, and lithium.

Antidepressants, especially selective serotonin reuptake inhibitors (SSRIs), which are widely used to manage depressive symptoms in mood disorders, have notable antibacterial properties [106]. For instance, sertraline demonstrates antimicrobial activity against both gram-positive (*Staphylococcus *and *Enterococcus *species) and gram-negative (*Pseudomonas aeruginosa* and *Klebsiella pneumoniae*) bacteria, whereas fluoxetine increases the risk of *Clostridium difficile* infection [107-109]. In addition, atypical antipsychotics, such as quetiapine and olanzapine, tend to reduce gut microbial diversity, notably affecting *Lachnospiraceae*, *Akkermansia*, and *Sutterella *taxa in rat models [110,111].

In contrast, lithium, a gold-standard treatment for bipolar mood disorder, enhances microbial diversity, particularly by increasing *Peptostreptococcaceae*, *Clostridiaceae*, and *Ruminococcaceae *families in animal studies [104,105]. In addition to lithium, anticonvulsants such as valproic acid also increase the abundance of *Clostridium *and related species, whereas lamotrigine shows antibacterial activity against gram-positive bacteria (*Bacillus *and *Staphylococcus aureus*), with limited effects on gram-negative strains [106,112].

Factors influencing gut microbiota

Exploring the factors that shape gut microbiota is crucial for understanding their impact on health, particularly in relation to mood disorders. Various biological, lifestyle, dietary, and environmental factors affect the composition and diversity of the gut microbiome, shedding light on its significance for human well-being.

Biological Factors

Starting with birth, the mode of delivery significantly affects an infant’s microbiome composition. For instance, vaginally delivered infants generally acquire microbiota resembling the vaginal microbiome, dominated by *Lactobacillus *and *Prevotella*, while cesarean-delivered infants acquire microbiota resembling the skin, with species such as *Streptococcus *and *Corynebacterium *[113]. Notably, vaginal delivery also promotes beneficial species such as *Bifidobacterium*, which are considered crucial for immune development [114]. In addition, as people age, especially the elderly, microbial imbalances (dysbiosis) become more common, which has been implicated in the development of conditions such as inflammatory bowel disease, obesity, diabetes, cardiovascular diseases, and neurodegenerative diseases. Research on how aging and microbiota interact, especially at disease onset, remains ongoing [115-117].

Lifestyle Factors

Lifestyle choices such as smoking, alcohol consumption, stress, sleep, and exercise heavily influence the gut microbiome. Aerobic exercise is hypothesized to have a beneficial effect on the gut microbiome, as it promotes a diverse microbiome with increased levels of beneficial *Firmicutes *and reduced amounts of *Bacteroides *compared to sedentary individuals, whereas excessive endurance exercise, such as in athletes, can cause dysbiosis [118-120]. Several mechanisms have been proposed to account for this alteration in the gut microbiota, including vagus nerve-mediated autonomic signaling, modulation of serotonin levels, and effects of neuroendocrines on the HPA axis [120].

Smoking introduces harmful compounds that can disrupt pH levels and lead to gut dysbiosis, whereas alcohol consumption can result in microbial imbalances linked to liver disease [121]. *Bacteroides *and *Enterococci *have been proposed to be present in individuals with alcoholic liver disease [121].

Furthermore, sleep patterns and circadian rhythms exhibit a bidirectional relationship with the gut microbiome; that is, disruption in one can lead to disruption in the other and vice versa. Altered sleep and circadian rhythms can alter the gut microbiota, with evidence showing that jet lag and poor sleep patterns are associated with gut changes [122].

Dietary Factors

The diet plays a key role in shaping the gut microbiome. For instance, breastfed infants harbor beneficial species such as *Lactobacillus *and *Bifidobacterium*, while formula-fed infants develop a different microbiome with predominant species such as *Enterococcus *and *Streptococcus *[123]. Consequently, breastfed infants have been shown to have a more stable gut microbiome with a robust immune response [124]. While in adults, plant-based diets like vegetarian and Mediterranean diets enhance the microbiome, increasing populations of anti-inflammatory microbes such as *Faecalibacterium*, which produce SCFAs. In contrast, diets high in animal products contribute to gut dysbiosis and inflammation, which may be linked to mood disorders and mental health conditions [125].

Stress

Stress exerts a negative influence on the gut microbiome through mechanisms such as the release of catecholamines and neuroendocrine hormones, altered vagal signaling, and gut hypoperfusion, which in turn produce reactive oxygen species (ROS). These changes can reduce gut motility, increase permeability, and promote inflammation and microbial translocation [126,127]. However, mild stressors such as cold exposure and moderate exercise can positively affect the microbiome, highlighting the complex relationship between stress and gut health and suggesting potential avenues for therapeutic intervention [128].

Interventions targeting gut microbiota

Diet and Probiotics

Dietary interventions and probiotics are emerging as complementary treatments for mood disorders; however, their current utility remains limited. Diets such as Mediterranean and anti-inflammatory diets are increasingly recommended for their ability to reduce inflammation and oxidative stress, which are factors linked to mood disorders [129,130]. Studies have highlighted that diets rich in fruits, vegetables, whole grains, and omega-3 fatty acids are correlated with lower incidences of depression and anxiety, whereas processed foods and refined sugars are associated with a higher risk of mood disorders [131]. These effects are likely mediated through the GBA, a bidirectional communication system between the gut microbiota and CNS, underscoring the impact of diet on mental health [132].

Probiotics, live microorganisms that confer health benefits, have shown promise in alleviating the symptoms of mood disorders by modulating gut microbiota and enhancing neurotransmitter production, such as serotonin and GABA, which are critical for mood regulation [133].

Psychobiotics

Psychobiotics, a specific class of probiotics targeting mental health, have demonstrated potential in reducing depressive symptoms, especially when used alongside traditional treatments, such as antidepressants and cognitive-behavioral therapy (CBT) [133]. Clinical trials suggest that probiotics can be particularly beneficial for individuals with GI disorders, such as IBS, which are often comorbid with depression and anxiety [134].

The future of treatment may involve personalized nutrition and tailoring of dietary recommendations based on individual microbiota profiles. Advances in microbiome sequencing could enable more targeted interventions by identifying microbial signatures linked to specific mood disorders [135]. Further research is needed to clarify the mechanisms through which probiotics and psychobiotics affect brain function, including their roles in neurotransmitter systems and inflammatory pathways [136]. The integration of dietary interventions, probiotic supplementation, and traditional therapies may offer novel strategies for managing mood disorders.

Precision Medicine Approaches

Precision medicine, an innovative strategy tailored to individual variability in genes, the environment, and lifestyle, holds significant promise for gut microbiota interventions. This approach customizes healthcare with medical decisions and treatments tailored to individual patients. In the context of the gut microbiota, precision medicine utilizes detailed microbial, genetic, and metabolic profiles to create personalized interventions aimed at modulating the gut microbiome and improving health outcomes [137].

One key strategy is personalized nutrition, which is based on an individual’s unique microbiome and metabolic profiles. These tailored nutrition plans promote beneficial bacterial growth while suppressing harmful strains. For instance, diets rich in specific fibers and prebiotics can enhance health-promoting bacteria, potentially reducing the risk of metabolic and inflammatory diseases [138]. Personalized probiotics, live microorganisms that confer health benefits, are integral to precision medicine. Identifying the most beneficial bacterial strains allows for the design of probiotics that can restore microbial balance and address gut-related health issues [139]. Genomic and metabolomic analyses are other methods that may play a crucial role in precision medicine.

Although still in the early stages, precision medicine approaches for gut microbiota demonstrate significant potential for enhancing health through targeted, personalized interventions. As research advances, these methods are expected to become vital for managing gut microbiota-related health issues.

## Conclusions

Gut microbiota influences mood disorders through pathways such as the GBA, immune modulation, and neurotransmitter production. Imbalances in gut bacteria have been linked to conditions like depression and bipolar disorder, while beneficial microbial byproducts, including SCFAs and neurotransmitters, contribute to brain function and emotional regulation. Although current research highlights a promising connection between gut health and mental wellness, several challenges remain.

Future studies should focus on larger, more diverse populations to establish causal links and assess the long-term efficacy of gut-targeted therapies. Individual variability in microbiome composition and response to treatment remains a major challenge, necessitating personalized approaches. Limitations in existing human studies, such as small sample sizes and short follow-up durations, hinder the translation of findings into clinical practice. Ethical and regulatory concerns regarding microbiota-based interventions, including fecal microbiota transplants and psychobiotics, also require careful consideration.
